# Whitewash for Ceilings

**Published:** 1890-06

**Authors:** 


### WHITEWASH FOR CEILINGS.

First wash the ceiling with clean water, frequently changed ; scrape the rough patches smooth, sweep with a broom, and stop all cracks and loose places. When this is done, clear away all rubbish and sweep the room. If the whitewash is to be made of lime, which is advisable after cases of illness, as it is a good purifier. a little preparation is required to prevent the lime-wash turning black, as it is apt to do. Mix into a stiff paste, with cold water, six balls of whiting ; to this add 2 pounds of very hot, but not boiling, size, and a small quantity of blue-black, ground fine, and let the whole get cold. By a little management the wash may be laid on without splashing, the method being, not to take too much at a time into the brush, or to jerk it at the end of the stroke. As a rule, ceilings and walls should be whitewashed at least once a year, and oftener, when necessary. For common work, a mixture of ½ bushel of lime, 1 lb. common salt, ½ lb. sulphate of zinc, and 1 gallon of sweet milk can be used. For brickwork exposed to damp, take ½ peck of well burnt lime, fresh from the kiln, slake with water, then add a sufficient quantity of water to reduce it to a paste, pass through a fine sieve ; add 1 gallon of clean white salt, which has been dissolved in boiling water, and a thin, smooth paste, also hot, made from 1 lb. fine rice flour, also ¼ lb. best glue. Mix these ingredients together, stir them well, and then add ¼ lb. best Spanish whiting, dissolved in 5 quarts boiling water. Stir again, and cover over to retain the heat and keep out dirt. Let it stand a week, when boil again, and apply hot. The above proportions will suffice to cover 40 square yards.

